# Effectiveness of acupuncture therapy as treatment for tinnitus: a randomized controlled trial^☆^

**DOI:** 10.1016/j.bjorl.2016.04.002

**Published:** 2016-04-30

**Authors:** Marcelo Yugi Doi, Simone Sayomi Tano, Adriane Rocha Schultz, Ricardo Borges, Luciana Lozza de Moraes Marchiori

**Affiliations:** a Rehabilitation Science Program Associate, Universidade Estadual de Londrina (UEL), Londrina, PR, Brazil; b Universidade Norte do Parana (UNOPAR), Londrina, PR, Brazil; c Faculdade de Medicina, Universidade Estadual de Londrina (UEL), Londrina, PR, Brazil; d Medical Residency Program Supervisor in Otolaryngology, Hospital Universitário de Londrina (UEL), Londrina, PR, Brazil

**Keywords:** Acupuncture therapy, Tinnitus, Quality of life, Randomized controlled trial, Rehabilitation, Terapia por acupuntura, Zumbido, Qualidade de vida, Ensaio clínico controlado aleatório, Reabilitação

## Abstract

**Introduction:**

Tinnitus is a subjective sensation of hearing a sound in the absence of an external stimulus, which significantly worsens the quality of life in 15–25% of affected individuals.

**Objective:**

To assess the effectiveness of acupuncture therapy for tinnitus.

**Methods:**

Randomized clinical trial (REBEC: 2T9T7Q) with 50 participants with tinnitus, divided into two groups: 25 participants in the acupuncture group and 25 participants in the control group. The acupuncture group received acupuncture treatment and the control group received no treatment. After a period of 5 weeks, they were called to perform the final evaluation and the control group received acupuncture treatment for ethical reasons.

**Results:**

A statistically significant result was found for the primary outcome, reducing the intensity of tinnitus, with *p* = 0.0001 and the secondary endpoint, showing improvement in quality of life, with *p* = 0.0001.

**Conclusion:**

Chinese scalp acupuncture associated with bilateral electroacupuncture demonstrated, in the short term, a statistically significant improvement by reducing the level of tinnitus intensity, as well as improving the quality of life of individuals with tinnitus.

## Resumo

**Introdução:**

O zumbido é a sensação subjetiva de ouvir um som na ausência de um estímulo externo e piora significativamente a qualidade de vida de 15%–25% das pessoas afetadas.

**Objetivo:**

Verificar a efetividade da terapia por acupuntura em indivíduos com zumbido.

**Método:**

Ensaio clínico aleatorizado com 50 participantes com zumbido, divididos em dois grupos: 25 participantes no Grupo da Acupuntura (GA) e 25 participantes do Grupo de Controle (GC). O GA recebeu tratamento com a craniopuntura chinês e o GC não recebeu nenhum tratamento. Após o período de 5 semanas, eles foram chamados para realizar a avaliação final e o GC recebeu tratamento de acupuntura por questões éticas de pesquisa.

**Resultados:**

Foi encontrado resultado estatisticamente significativo para o desfecho primário, reduzindo a intensidade do zumbido, com *p* = 0,0001 e o desfecho secundário, apresentando melhora na qualidade de vida, com *p* = 0,0001.

**Conclusão:**

A craniopuntura chinesa associada à eletroacupuntura bilateral nos mostrou no curto prazo, uma melhora estatisticamente significativa, reduzindo o nível de intensidade do zumbido, bem como melhorando a qualidade de vida dos indivíduos com zumbido.

## Introduction

Tinnitus is a symptom defined as the perception of sound without the presence of an external sound source.^1^

It is estimated that approximately 5–15% of the population has of some type of tinnitus and, although it can occur at any age, it is more prevalent among the elderly (mainly in those aged between 60 and 69 years) than in young adults.^2^ In a study on the incidence of tinnitus, Nondahl et al.^3^ followed a group of 2922 adult and elderly individuals, aged 48–92 years, for 10 years. During the first 5 years of follow-up in this same group, they observed that the incidence of tinnitus in the assessed population was 5.7%.^4^ After 10 years, the authors found that the incidence had more than doubled, reaching 12.7%.^3^

In Brazil, it is estimated that 17% of the population is affected by tinnitus, i.e., more than 28 million Brazilians.^5^ Santos et al.^6^ evaluated 406 patients in a 6 month period and found that 58% had a tinnitus complaint; of these, 68% were females and 32% males. In a study carried out by Gibrin et al.^7^ in 2012, the authors evaluated 519 individuals of both genders with a median age of 69 years and found a prevalence of 42.77% for tinnitus complaints.

It is currently believed that tinnitus occurs as a result of the dynamic interaction of several centers of the nervous and the limbic system, and that cochlear alterations and/or lesions are the precursors of this process, causing imbalance in the lower auditory pathways, resulting in abnormal neuronal activity, further enhanced by the central nervous system, and finally perceived as tinnitus.^8^

Several etiologies have been proposed, including otologic, dental, neurological, psychiatric diseases, cervical spine and metabolic disorders, as well as others related to the intake of drugs, caffeine, alcohol, and tobacco.^9^

However, the physiopathological mechanisms of tinnitus are not well-defined, and therefore the treatment remains a major challenge to date. Symptom subjectivity and the wide etiological variety, often seen in the same patient, make it difficult to obtain good results.^9^ Moreover, currently, no one specific treatment, including drug therapy, is considered effective in treating the symptoms of tinnitus.^10^

Complementary and alternative medicine has often been used to treat tinnitus, and acupuncture is one of the most often-used options.^10^ Acupuncture is a therapeutic method that involves the insertion and manipulation of needles into the body. The treatment of tinnitus by acupuncture has been widely described in books^11^, ^12^; however, the scientific literature still lacks studies supporting its therapeutic effectiveness. Studies have shown that stimulation performed with needles promotes introduction of an electrical charge that triggers action potentials in order to rebalance the system.^13^, ^14^

Chinese scalp acupuncture is a contemporary acupuncture technique that has only 40 years of history. It integrates traditional Chinese insertion methods with Western medical knowledge of the cerebral cortex, and has been proven to be a very effective technique for the treatment of several diseases of the central nervous system,^15^ as well as for alleviating the symptoms of tinnitus.^9^

In 2000, Park et al.^16^ performed a systematic review and identified 36 publications on the subject, but only 6 were randomized controlled trials. The authors mentioned that the prescription of points was heterogeneous and that results were controversial. Therefore, they suggested that future studies on this subject are necessary and should be performed according to the highest methodological standards.

Hence, this study aimed to determine the effectiveness of an acupuncture program as a therapeutic procedure in patients with tinnitus.

## Methods

This was a randomized clinical trial (RCT) that included individuals of both genders, aged between 50 and 85 years, with moderate continuous tinnitus for at least 1 year, in whom tinnitus interfered with the quality of life measured by the Tinnitus Handicap Inventory (THI) questionnaire.^17^

Exclusion criteria were: individuals with active cardiovascular disease, those using pacemakers or metal implants, and those who had a score at the THI questionnaire showing only slight or mild degrees of interference with quality of life. In addition, subjects who were participating in other treatment programs for tinnitus were excluded.

All participants signed an informed consent. The study was approved by the Ethics Committee in Research (number 95,055) and was registered in the Brazilian clinical trial register (REBEC: 2T9T7Q).

The study participants were recruited through interviews on the local television programs of three local stations, which introduced the research project, informed the population about the tinnitus, and provided recommendations to those interested in participating in the study. Through these talk shows, 76 phone calls were received from individuals that met the study criteria and were called for evaluation.

**Figure 1:**
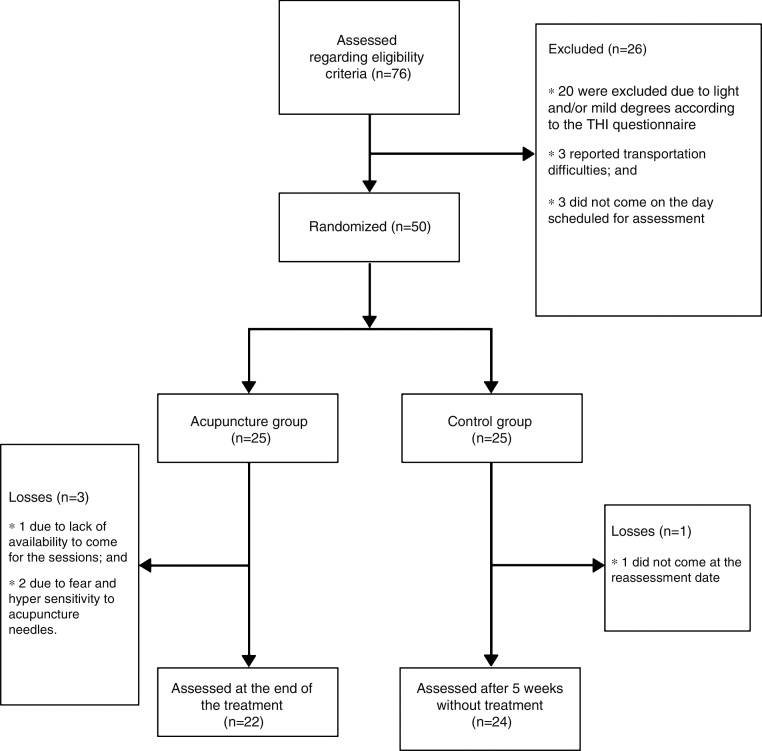
Flowchart.

Of these 76 assessed patients, 20 were excluded due to light and/or mild degrees according to the THI questionnaire; 3 reported transportation difficulties, and 3 did not appear for the evaluation appointment. Therefore, a total of 50 individuals of both genders, aged between 50 and 85 years, who were not undergoing another treatment for tinnitus and who had moderate to severe scores at the THI questionnaire, as shown in the flowchart (Fig. 1), were included in the study.

The 50 participants were randomly divided into two groups. Randomization was carried out with the aid of computerized table of random numbers created by a Microsoft Excel spreadsheet.

The randomization result was kept in sealed, opaque envelopes to ensure selection concealment. One group was termed the acupuncture group (AG), which included 25 participants who received treatment through an acupuncture program; the control group (CG) included 25 participants who received no treatment. Fig. 1 shows the flowchart of participants’ enrollment and randomization. After the start of the interventions, three participants from the AG withdrew from the study, one due to lack of availability to attend the sessions and two due to fear and hypersensitivity to the acupuncture needles, thus totaling 22 individuals in the AG. One participant from the CG also withdrew from the study, after failing to attend a revaluation consultation.

Evaluations of AG participants were performed before and after 5 weeks of intervention, by a previously trained researcher who was blinded to treatment or control group allocation. Evaluations in the CG were performed at enrollment and after a 5 week interval without any treatment, by a previously trained researcher who was blinded to treatment or control group allocation.

A case history form was completed to characterize the sample. It contained information on gender, age, presence or absence of tinnitus, unilateral or bilateral tinnitus, the duration of tinnitus manifestation, type of tinnitus (continuous or intermittent), presence or absence of dizziness, ear surgery, history of exposure to occupational noise, diabetes or high blood pressure, pacemaker use, metallic implants, medications used, and whether the patient was undergoing or had undergone any kind of treatment for tinnitus and if so, for how long, based on the Katz protocol for anamnesis.^18^

Hearing assessment was performed with an otoscopic evaluation of the external ear canal and tympanic membrane, and with pure tone audiometry, considered the gold standard to assess auditory threshold in adults.^19^ Hearing loss was classified according to Silman and Silverman^20^ and the classification of Lloyd and Kaplan was used to grade the degree of hearing loss.^21^

### Visual analog scale (VAS)

**Figure 2:**
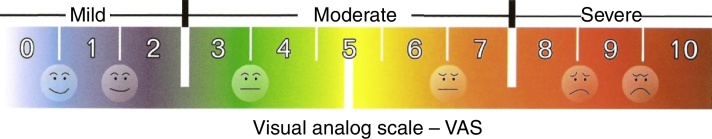
Visual analog scale.

To assess the level of intensity of the tinnitus, a visual analog scale (VAS) was used (Fig. 2), consisting of a visual graphic tool to determine the volume level or intensity or discomfort caused by the tinnitus, on a scale of 0–10. The patient is asked to give a score from 0 to 10 to the intensity generated by the tinnitus, where 0 represents total absence of tinnitus symptom and 10 indicates a maximum intensity of tinnitus symptom, requiring going to a hospital to receive care.^14^, ^22^ The VAS was applied at the initial and final assessments, both in the AG and the CG.

### THI questionnaire

The THI is a questionnaire developed by Newman et al.^17^ in 1996, and consists of 25 questions with a score ranging from 0 to 100, where the higher the score, the greater the impact of tinnitus on patient quality of life. It is a rapid measurement, easy to apply and interpret. It has been broadly used in the clinical setting to assess patients with tinnitus.

The three main items assessed by the THI are: functional reactions to tinnitus, such as difficulty concentrating and antisocial tendencies; emotional reactions to tinnitus, such as anger, frustration, irritability, depression; and catastrophic reactions to tinnitus, such as despair, hopelessness, fear of severe disease, loss of control, and incapacity to cooperate.

THI is currently one of the most accepted methods for tinnitus assessment, having been validated in many consensuses. It was translated and culturally adapted to be used in the Brazilian population in 2005^23^ and its validation and reproducibility were carried out in 2006.^24^

### Intervention

The intervention lasted 5 weeks, with a twice-a-week frequency, totaling ten sessions, with each session lasting approximately 40 min. The acupuncture program was conducted by a specialist and supervised by the researcher. Participants were instructed to report any complaint, whether or not associated to the treatment. Both groups were instructed not to participate in any other tinnitus treatment program throughout the study period.

**Figure 3:**
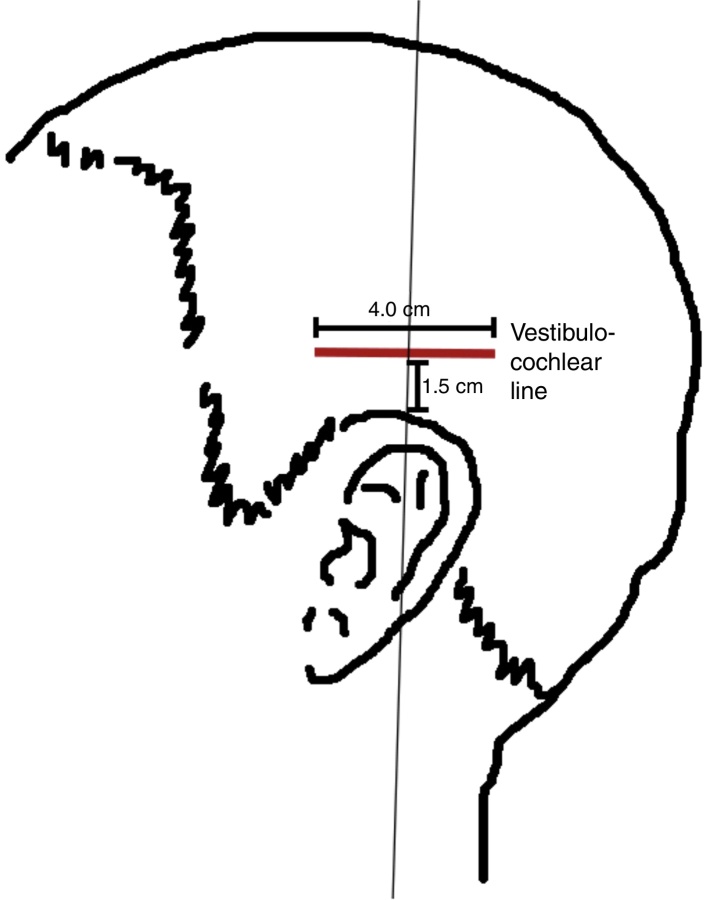
Vestibulocochlear line according to Chinese scalp acupuncture.

In the AG, the participants received treatment that followed an acupuncture program using the Chinese scalp acupuncture technique associated with electrostimulation, bilaterally in the vestibulocochlear line (Fig. 3). This line is located 1.5 cm above the ear apex in a horizontal line segment corresponding to 4 cm.^12^

In the control group (CG), the participants did not receive acupuncture treatment. These participants were evaluated and then waited 5 weeks, after being told not to receive or undergo any treatment for tinnitus during this period, including drug treatment, and to continue with their normal daily activities. After the 5 week period, they were called in to undergo the final evaluation. It was only after the final assessment that these participants received acupuncture treatment, for ethical reasons. The acupuncture treatment program was the same to which the AG was submitted.

### Statistical analysis

Data were analyzed descriptively and analytically. Numerical variables were observed regarding the distribution of normality using the Shapiro–Wilk's test. As the assumption of normality was not met, the data are shown as median (Md) and its quartiles (1st–3rd). Categorical variables are shown as absolute and relative frequencies.

The Mann–Whitney *U* test was used to compare between the groups, and the Wilcoxon test was used to assess within each group.

All analyses were performed by intention to treat using the SPSS v. 15.0 software and statistical significance was set at 5% (*p* ≤ 0.05).

## Results

Seventy-six subjects were evaluated regarding the study eligibility criteria. Of these, 26 (34.2%) were excluded and 50 (65.8%) participated in the study. Only 46 participants (92%) completed the treatment. Descriptive characteristics of the sample are shown in Table 1.

**Table 1:** Characteristics of the participants assessed before treatment.

	Groups			
	AG (*n* = 22)	*p*	CG (*n* = 23)	*p*
*Gender, n (%)*				
Male	8 (36.3)		10 (43.4)	
Female	14 (63.7)		13 (56.6)	
*Age (years)*	62 [57–67.5]		60 [54.5–62]	
*Hearing (n; %)*				
Bilateral loss	17 (77.3)		18 (78.3)	
Unilateral loss	3 (13.7)		2 (4.3)	
No loss	2 (9)		4 (17.4)	
*HL Grade (n)*	RE/LE		RE/LE	
NL with decrease	7/6		9/9	
Mild	7/6		4/5	
Moderate	2/5		5/4	
Severe	1/2		1/1	
*Initial VAS*	8 [7–9]	0.331	8 [7.5–9.5]	0.331
*Inicial THI*	56 [44–65.5]	0.176	58 [48–76]	0.176

M, male; F, female; HL, hearing loss; NL, normal; RE, right ear; LE, left ear; values shown as median [1st quartile; 3rd quartile]; Mann–Whitney *U* test considering *p* > 0.05; AG, acupuncture group; CG, control group; Initial VAS, initial visual analog scale; Initial THI, initial Tinnitus Handicap Inventory questionnaire.

Of the 46 participants, 22 were allocated to the AG and of these, 8 were males and 14 females, with a median age of 62 years; 17 had bilateral sensorineural hearing loss, 3 had unilateral sensorineural hearing loss, and 2 did not have hearing loss. Twenty-four participants were allocated to the CG, of which 10 were males and 13 females, with a median ge of 60 years; 18 had bilateral sensorineural hearing loss, 2 had unilateral sensorineural hearing loss, and 4 did not have hearing loss.

Of the 90 ears assessed, the vast majority, 74 (82.2%) showed sensorineural hearing loss; of these, 31 (34.4%) had normal degree, with a decrease in acute frequencies, 23 (25.5%) were mild, 16 (17.7%) were moderate, 4 (4.4%) were severe, and none were profound.

Concerning the primary outcome, intensity level of tinnitus measured by the VAS, there was a statistically significant difference, *p* = 0.0001 (Table 2), when comparing between the AG assessment at baseline and after treatment. The data showed a reduction of almost 50% in tinnitus intensity when comparing AG and CG from baseline to treatment with acupuncture.

**Table 2:** Primary and secondary endpoint, and comparison between groups.

	Acupuncture group (*n* = 22)	Control group (*n* = 24)	*p*
Initial VAS	8 [7–9]	8 [7.5–9.5]	0.580
Final VAS	4 [3–6]	8 [8–10]	0.0001
*p*	0.0001	0.168	

Initial THI	56 [44–65.5]	58 [48–76]	0.331
Final THI	28 [8–55.5]	68 [46–76]	0.0001
*p*	0.0001	0.943	

Md, median; 1st–3rd Q, first and third quartiles; VAS, visual analog scale; THI, Tinnitus Handicap Inventory questionnaire; Mann–Whitney *U*, *p* < 0.05.

There was no statistically significant difference when comparing the results from the data evaluated at baseline and after 5 weeks in the CG, with *p* = 0.168 (Table 2).

To assess the results of the secondary outcome (improvement in quality of life as measured by the THI questionnaire) the THI scores at baseline, were compared with THI after treatment in the AG, and a statistically significant difference was observed (*p* = 0.0001; Table 2), with the median data revealing a decrease from grade 4 (severe interference in the quality of life) to grade 2 (mild interference in quality of life) of tinnitus influence on quality of life, whereas in the CG, there was no statistically significant difference, with *p* = 0.943 (Table 2).

When comparing the AG and CG regarding the values of the initial VAS scores and the initial THI questionnaire, obtained in the assessment before the start of the treatment, the results showed no statistically significant difference, with *p* = 0.580 and *p* = 0.331, respectively. However, when comparing the AG and CG regarding the values of final THI and final VAS obtained after the treatment, the results showed to be statistically significant, with *p* = 0.0001 and *p* = 0.0001, respectively (Table 2).

## Discussion

The treatment of tinnitus with AC has been widely described in TCM. The scientific literature, however, lacks studies to demonstrate its effectiveness as a treatment option. AC is a holistic form of treatment tailored to each individual. Thus, protocols with adequate standardized methods that meet the principles of TCM and modern Western medicine are difficult to create.^9^

This study aimed to compare the effectiveness of an acupuncture program for the treatment of patients with tinnitus. The results indicate that treatment with acupuncture improves the perception of tinnitus, decreases the intensity level, and improves the quality of life of individuals with tinnitus. That differs from most literature results,^25^, ^26^ in which the studies reported no significant difference between treatment with acupuncture and placebo.

Only a few studies reported a significant effect of acupuncture for the treatment of tinnitus; however, these effects were significant only for immediate relief.^9^

One factor that can explain this difference is the number of treatment sessions performed in these studies. Most of them had a treatment program with fewer than tem acupuncture sessions, and two of these studies used a single session, which may be insufficient to obtain a positive clinical outcome.^9^, ^25^, ^26^, ^27^

Another bias inherent to these studies was evaluated based on the description of the participant randomization methods. Most of the included studies had a high risk of bias. Low-quality trials are more likely to overestimate the effect size. Moreover, none of the studies used a statistical calculation indicating the power of study sizes, and samples were obtained by convenience, and thus were nonrepresentative.^28^, ^29^

Axelsson et al.,^26^ in a study of 20 patients with tinnitus triggered by noise, found no significant difference in symptoms between the placebo and control groups. However, they observed that the study participants showed improvement in sleep quality, blood circulation, and muscle relaxation.

In the present study, these aspects also showed improvement, and the final result of the THI questionnaire indicates that the participants in the control group had a severe degree of interference with quality of life, which often shows sleep pattern alterations and interference with the activities of daily life. The participants of the AG showed improvement in the degree of interference with quality of life to mild after treatment with acupuncture, easily masked by the environment and also easily forgotten in the presence of usual noises during activities of daily living.

In another study by Jeon et al.,^30^ the long-term effect of an acupuncture program for the treatment of tinnitus was assessed. Thirty-three subjects were divided into two groups: one group treated with acupuncture and another group with sham acupuncture. The acupuncture treatment program consisted of ten sessions, twice a week, lasting 5 weeks. The participants were evaluated prior to treatment, at the end of treatment, and at 3 months of follow-up. The assessment tools used were the VAS and THI questionnaire. In this study, there was no statistical difference in any outcome between the real acupuncture and sham acupuncture.

However, the THI questionnaire scores showed a significant improvement at the end of treatment when compared to baseline, and this effect was maintained throughout the follow-up period (after 3 months); however, this result was not observed in the sham acupuncture group.

When compared through VAS, both the acupuncture and the sham acupuncture groups showed significant improvement from baseline to 3 months of follow-up. However, there was no statistical difference in any outcome between the treatment group and the sham group. The acupuncture group showed statistical significance only for the mean percentage variation in VAS, compared to sham acupuncture, from the start of treatment until 3 months of follow-up, with *p* = 0.019.

As in the study by Jeon et al.,^30^ the present study shows an acupuncture therapy program consisting of ten sessions, twice a week, with a total duration of 5 weeks. This study also used the VAS tool to assess tinnitus intensity. This tool is widely used to measure the volume reported by tinnitus patients,^25^, ^31^, ^32^ mainly due to the fact that it is fast and easy to apply and, therefore, it is easy to compare results between groups.

A statistically significant difference was found in the variation of the VAS scores between the AG and CG, and between the initial and final evaluations, with *p* = 0.0001.

Similarly to the VAS tool, the THI questionnaire is also one of the most often used tools in international literature to assess the interference of tinnitus on quality of life. The THI questionnaire is currently one of the most accepted methods for assessing tinnitus, having been validated in several consensuses.^32^ This study also used the THI questionnaire to assess the quality of life of patients with tinnitus, as it has been used in several other studies.^32^, ^33^

The present study demonstrated statistically significant differences in the THI questionnaire scores between the AG and CG, between the initial and final evaluation.

This study showed that there is effects of tinnitus symptom relief as a result of Chinese scalp acupuncture. Other non-specific factors unrelated to the vestibulocochlear line of Chinese scalp acupuncture may be related to this effect, such as induction by the patient's subjectivity and the increased attention given by doctors to the study patients.^9^

The significant levels of improvement of the effect justify the use of this technique. This study shows that the technique is safe and does not cause any side effects for patients. However, more studies are required to establish other possible effects of Chinese scalp acupuncture on the auditory system.

### Study limitations

Among the limitations of this study is the lack of follow-up. Thus, it was not possible to assess whether the acupuncture program had a medium- or long-term effect on perception of tinnitus, decrease in intensity, and improved quality of life of individuals with tinnitus, and whether the effectiveness obtained was palliative or therapeutic.

Another limitation was the possibility of the placebo effect of the acupuncture intervention, since the control group did not undergo any intervention to obviate this possibility; thus the effect of placebo treatment cannot be ruled out in cases of tinnitus.

Furthermore, although this study followed the sample size calculation, it would be prudent to perform a study with a larger number of participants, i.e., to ensure a safety margin considering possible losses that occurred throughout the study.

### Implications for future studies

More studies with high methodological quality and low risk of bias are required to assess the effect of acupuncture on the treatment of tinnitus. The rules of the Consort statement must be followed and described in detail, as well as the sample size calculation, so that a systematic review with meta-analysis can be performed.

## Conclusion

The patients showed a significantly improved tinnitus perception. The technique of Chinese scalp acupuncture associated with bilateral electroacupuncture showed a statistically significant improvement in reducing tinnitus intensity level, as well as improving the quality of life in patients with tinnitus in the short term.

## Conflicts of interest

The authors declare no conflicts of interest.

## References

[1] Pinto P.C.L., Sanchez T.G., Tomita S. (2010). Avaliação da relação entre severidade do zumbido e perda auditiva, sexo e idade do paciente. Braz J Otorhinolaryngol.

[2] Norena A.J. (2011). An integrative model of tinnitus based on a central gain controlling neural sensitivity. Neurosci Biobehav Rev.

[3] Nondahl D.M., Cruickshanks K.J., Wiley T.L., Klein B.E., Klein R., Chappell R. (2010). The ten-year incidence of tinnitus among older adults. Int J Audiol.

[4] Nondahl D.M., Cruickshanks K.J., Wiley T.L., Klein R., Klein B.E., Tweed T.S. (2002). Prevalence and 5-year incidence of tinnitus among older adults: the epidemiology of hearing loss study. J Am Acad Audiol.

[5] Possani L.N.A. (2006).

[6] Santos T.M.M., Branco F.C.A., Rodrigues P.F., Bohsen Y.A., Santos N.I. (1999). Proceedings of the VI international tinnitus seminar.

[7] Gibrin P.C.D., Melo J.J., Marchiori L.Ld.M. (2013). Prevalência de queixa de zumbido e prováveis associações com perda auditiva, diabetes mellitus e hipertensão arterial em pessoas idosas. CoDAS.

[8] Moreira M.D., Marchiori L.Ld.M., Costa Vd.S.P., Damasceno E.C., Gibrin P.C.D. (2011). Zumbido: possível associac¸ão com alterações cervicais em idosos. Arq Int Otorrinolaringol.

[9] Okada D.M., Onishi E.T., Chami F.I., Borin A., Cassola N., Guerreiro V.M. (2006). Acupuncture for tinnitus immediate relief. Braz J Otorhinolaryngol.

[10] Kim J.I., Choi J.Y., Lee D.H., Choi T.Y., Lee M.S., Ernst E. (2012). Acupuncture for the treatment of tinnitus: a systematic review of randomized clinical trials. BMC Complement Altern Med.

[11] Maciocia G. (2007).

[12] Yamamura Y. (2004).

[13] Pomeranz B., Chiu D. (1976). Naloxone blockade of acupuncture analgesia: endorphin implicated. Life Sci.

[14] Azevedo R.Fd., Chiari B.M., Okada D.M., Onishi E.T. (2007). Efeito da acupuntura sobre as emissões otoacústicas de pacientes com zumbido. Braz J Otorhinolaryngol.

[15] Hao J.J., Cheng W., Liu M., Li H., Lu X., Sun Z. (2013). Treatment of multiple sclerosis with Chinese scalp acupuncture. Glob Adv Health Med.

[16] Park J., White A.R., Ernst E. (2000). Efficacy of acupuncture as a treatment for tinnitus: a systematic review. Arch Otolaryngol Head Neck Surg.

[17] Newman C.W., Jacobson G.P., Spitzer J.B. (1996). Development of the Tinnitus Handicap Inventory. Arch Otolaryngol Head Neck Surg.

[18] Katz J. (1989).

[19] Gorga M.P., Neely S.T., Dorn P.A. (1999). Distortion product otoacoustic emission test performance for a priori criteria and for multifrequency audiometric standards. Ear Hear.

[20] Silman S., Silverman C.A. (1997).

[21] Lloyd L.L., Kaplan H. (1978).

[22] Azevedo A.Ad., Oliveira P.Md., Siqueira A.Gd., Figueiredo R.R. (2007). Análise crítica dos métodos de mensuração do zumbido. Braz J Otorhinolaryngol.

[23] Ferreira P.É.A., Cunha F., Onishi E.T., Branco-Barreiro F.C.A., Ganança F.F. (2005). Tinnitus Handicap Inventory: adaptação cultural para o Português brasileiro. Pró-Fono R Atual Cient.

[24] Schmidt L.P., Teixeira V.N., Dalligna C., Dallagnol D., Smith M.M. (2006). Adaptação para língua portuguesa do questionário Tinnitus Handicap Inventory: validade e reprodutibilidade. Braz J Otorhinolaryngol.

[25] Marks N.J., Emery P., Onisiphorou C. (1984). A controlled trial of acupuncture in tinnitus. J Laryngol Otol.

[26] Axelsson A., Andersson S., Gu L.D. (1994). Acupuncture in the management of tinnitus: a placebo-controlled study. Audiology.

[27] Tan K.Q., Zhang C., Liu M.X., Qiu L. (2007). Comparative study on therapeutic effects of acupuncture, Chinese herbs and Western medicine on nervous tinnitus. Zhongguo Zhen Jiu.

[28] Schulz K.F., Chalmers I., Hayes R.J., Altman D.G. (1995). Empirical evidence of bias. Dimensions of methodological quality associated with estimates of treatment effects in controlled trials. JAMA.

[29] Day S.J., Altman D.G. (2000). Statistics notes: blinding in clinical trials and other studies. BMJ.

[30] Jeon S.W., Kim K.S., Nam H.J. (2012). Long-term effect of acupuncture for treatment of tinnitus: a randomized, patient- and assessor-blind, sham-acupuncture-controlled, pilot trial. J Altern Complement Med.

[31] Furugard S., Hedin P.J., Eggertz A., Laurent C. (1998). Acupuncture worth trying in severe tinnitus. Lakartidningen.

[32] Figueiredo R.R., Azevedo A.A.D., Oliveira P.D.M. (2009). Análise da correlação entre a escala visual-análoga e o Tinnitus Handicap Inventory na avaliação de pacientes com zumbido. Braz J Otorhinolaryngol.

[33] Mathias KdV., Mezzasalma M.A., Nardi A.E. (2011). Prevalência de transtorno de pânico em pacientes com zumbidos. Revista Psiquiatr Clín.

